# Effects of sodium alginate capsules as 3D scaffolds on hormones and genes expression in preantral follicles of mice compared to 2D medium: An experimental study

**DOI:** 10.18502/ijrm.v13i7.7369

**Published:** 2020-07-22

**Authors:** Cyrus Jalili, Fuzieh Khani Hemmatabadi, Kamran Mansouri, Mehrdad Bakhtiyari

**Affiliations:** ^1^Department of Anatomical Sciences, Medical Biology Research Center, Kermanshah University of Medical Sciences, Taghbostan, Kermanshah, Iran.; ^2^Medical Biology Research Center, Kermanshah University of Medical Sciences, Kermanshah, Iran.; ^3^Department of Anatomy, School of Medicine, Iran University of Medical Sciences, Tehran, Iran.

**Keywords:** Follicle, Three-dimensional culture, Sodium alginate.

## Abstract

**Background:**

The improvement of in vitro maturation methods, which can activate the preantral follicle growth, plays a crucial role in the production of mature oocytes in reproductive technology.

**Objective:**

To evaluate the different concentrations of 3D scaffolds of sodium alginate on hormones and gene expression in mice preantral follicles.

**Materials and Methods:**

Immature female BALB/c mice (12-14 days) were sacrificed. The follicles were removed mechanically and transferred into α minimal essential medium with 5% fetal bovine serum. The preantral follicles were incubated with different concentrations of sodium alginate (0.25%, 0.5%, and 1%) and 2D medium for 12 days. The follicles were examined for antral formation following the 10th day and the diameter on days 6${}^{\mathrm{th}}$ and 12${}^{\mathrm{th}}$. The levels of hormones (AMH, androstenedione, 17β-estradiol, and progesterone) and the expression of genes (*CYP11a1*, *CYP17a1*, *CYP19a1*, *AMH*, and *GnRH*) at the end of the 12${}^{\mathrm{th}}$ day.

**Results:**

Maximum follicle diameter and highest percentage of antrum formation were related to 0.5% concentration (p = 0.00). The levels of hormones in different doses of sodium alginate were increased significantly compared to the control group (p = 0.00). The highest and lowest levels of these hormones were related to 0.5% concentration and 2D medium, respectively. The highest level of genes expression was observed in 0.5% sodium alginate, which showed a significant increase compared to the control group (p = 0.00).

**Conclusion:**

Proper concentration of alginate hydrogel increases follicle growth, causes follicle maturation, produces steroid hormones, and increases appropriate expression of steroidogenesis-related genes.

## 1. Introduction

The growth and development of follicles and in vitro maturation (IVM) of the oocytes are new methods used in Assisted Reproductive Technology (ART) (1). In the last two decades, numerous culturing methods have been designed for in vitro growth and maturation of follicles and oocytes. These technologies can help follicles to grow in the culture medium and to mature the oocyte for increasing the fertility opportunity in patients under chemotherapy and radiotherapy procedures. These interventional methods cause full or partial destruction of the follicular reserve (2). In ovulation induction during ART, more follicles grow; besides the patient is at a risk of ovarian hyper stimulation syndrome (OHSS) (3). Thus, the application of follicles without ovulation stimulation and the IVM therapy are known as appropriate methods to recruit immature cells and prevent the OHSS (4).

The use of IVM for immature oocytes is an appropriate procedure for patients especially with polycystic ovary syndrome. The IVM can reduce ovulation induction and the risk of OHSS as one of the clinical emergencies (5). Thus, these artificial conditions in IVM medium must compensate to the inappropriate conditions available in the patient group, these created artificial environments are similar to that of the normal follicular conditions that stimulate and mature the oocytes to grow and develop, and prevent the adverse effects of the culture medium on the oocyte (6). The encapsulated cells in hydrogels cause the uniform distribution of these cells in the gel matrix. The hydrogel permeability is responsible for the proper diffusion of oxygen, nutrition, and biochemical stimuli into the environment. Besides, the physical property of elasticity of hydrogel is considered as a type of physical stimulus (7). One of the important challenges in encapsulated ovarian follicles is the selection of appropriate biomaterials. Alginate is one of the most widely used hydrogels in tissue engineering (3). Sodium alginate is a natural polymer driven from the brown seaweed. It consists of a-L-Guluronic acid and b-D-Mannuronic acid polymers that form the alginate hydrogel in the presence of calcium without any need for special environments like chemicals, light, or temperature (8).

In the present study, three-dimensional (3D) culture medium was used to achieve in vitro conditions similar to in vivo to compare the development of preantral follicles in 3D culture medium containing alginate with concentrations of 0.25%, 0.5%, and 1% and two-dimensional (2D) culture media and also to design an optimal culture medium for follicular maturation with high efficacy. One of the crucial challenges is the improvement of the culture medium. The purpose of this study was to investigate the effect of culture medium changes on the developmental potential of follicles and the effects of different concentrations of 3D sodium alginate scaffolds on genomic alterations of follicles.

## 2. Materials and Methods

### Study animals and the ovaries

In this study, immature female BALBc/mice (12-14 days) with preantral follicles were selected. They were kept in photocycle 12 hr dark light in an environment with a temperature of 23°C and the humidity of 44%. The animals were sacrificed by cervical dislocation. Their abdomen was longitudinally cut; the ovaries were removed and placed in α minimal essential medium (α-MEM) in the presence of 5% fetal bovine serum (FBS). Residual surrounding tissues were dissected by an insulin needle. In order to evaluate the follicles at different times, the ovaries were placed in a culture medium in an incubator to isolate the follicles.

### Isolation of preantral follicles

In this regard, the mechanical method was used with a 29G needle attached to a 1 ml-insulin syringe by a stereomicroscope. The preantral follicles with a normal structure and a diameter of 100-150 μm were selected. In the present study, the sample size of 80 follicles was grouped as follows: control group (2D culture media) and three treatment groups with different concentrations of sodium alginate (0.25%, 0.5%, 1%).

### Preparation of sodium alginate hydrogel

All materials were obtained from the Sigma-Aldrich Co. The sodium alginate with concentrations of 0.25%, 0.5%, and 1% was initially mixed with phosphate buffer saline to prepare the alginate hydrogel. The impurities of solution were cleaned by 0.5 gr of activated charcoal per gram of sodium alginate. The obtained material was filtered with 0.22-μm Millipore Filter and kept in a refrigerator (at 4°C). The isolated follicles were prepared for encapsulation with sodium alginate hydrogels (0.25%, 0.5%, and 1%) following washing in the culture medium.

### Encapsulation procedure and 3D culture of preantral follicles

To encapsulate the preantral follicles, they were transferred to 5 µl of sodium alginate droplets with different concentrations. The droplets were also transferred into a calcium bath (containing 140 mM of CaCl${}_{2}$ and 50 mM of NaCl) for 2 min using a micropipette to produce the calcium bonding of the hydrogel. Next, the alginate hydrogel droplets were gathered and rinsed with the medium. Under the microscopy investigation, only the encapsulated follicles available in the center of hydrogel were selected for culture. The cultivation of isolated follicles is a crucial process due to the absence of blood supply to granulosa cells.

In this method, whole follicles can be cultured independently, measured during the culturing period, and examined for any changes. Each encapsulated follicles were transferred into 40-µl droplets of the under-oil culture medium in 96-well plates for culture. In this regard, 2D and 3D culturing methods were performed using α-MEM medium supplemented with 1% Insulin-transferrin-selenite (ITS), 100 IU/ml of penicillin, 100 μg/ml of streptomycin, 100 IU/ml of recombinant follicle-stimulating hormone (rFSH), 5% FBS and 10 ng/ml of recombinant epidermal growth factor (rEGF). The medium was kept at 2-8°C for a week (9). The follicles were cultured in a humidified incubator at 37°C and 5% CO${}_{2}$ for 12 days. Half of the culture medium was exchanged with a fresh medium for a day and stored at -80°C to evaluate the hormonal changes.

### Follicle retrieval

For RNA isolation or oocyte maturation, the encapsulated follicles were removed from the hydrogel substrate on day 12. For this purpose, in a 3D culture system, the follicles were removed from the hydrogel following the adding of 5 mg of ethylene glycol tetra acetic acid (EGTA) to the culture medium for 5 min at 37°C. The required follicles with a minimum amount of medium were transferred to the cleaned test tubes to isolate the RNA. Finally, they were cryopreserved at -80°C for future analysis.

### Measurement of follicles diameter 

**Figure 1:**
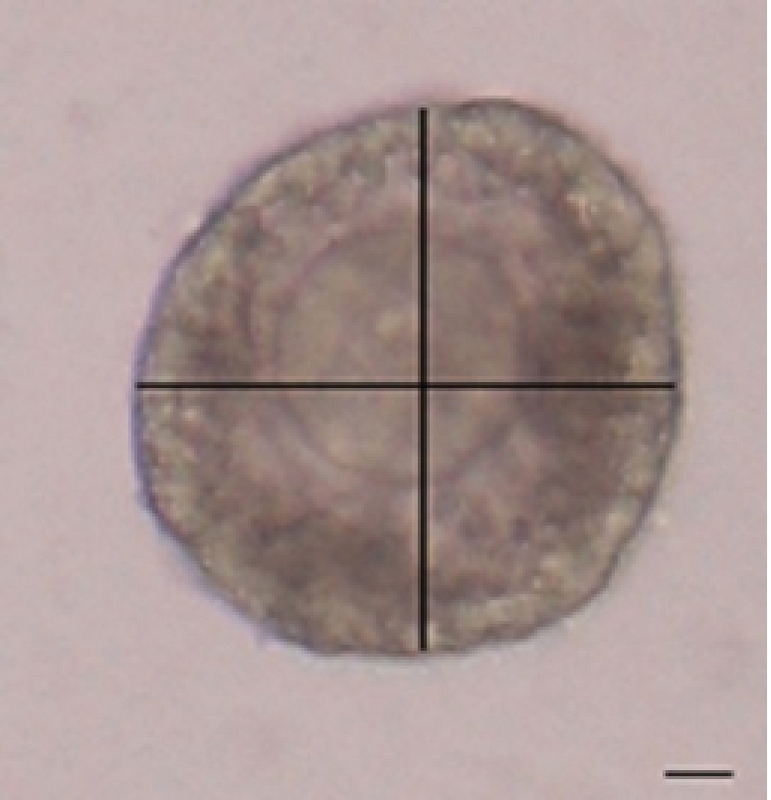
Measurement of follicle diameter. Bar = 100 μm.

Since the measurement of follicle diameter is possible from day 4^th^ onward (due to the separation of theca and granulosa cells and formation of irregular follicles), thus this method was evaluated in the 2D culture at 4 (Figure 1). The diameter of the spherical shape of follicles was measured by the mean size of two perpendicular diameters (µm) on days 2, 6, and 12 (100x).

### Evaluation of morphological changes and survival rate of follicles

At the end of the 12${}^{\mathrm{th}}$ day, the follicles were examined morphologically. The arrest of granulosa cells proliferation and follicular growth, early ovulation, and dark follicles were regarded as degenerated follicles. At the end of the 10${}^{\mathrm{th}}$ day, the antrum formation was evaluated in the follicles and its survival rate was calculated in comparison with the surviving follicles.

### Steroid assays

The levels of androstenedione, 17β-estradiol, progesterone, and AMH hormones extracted from the follicles in culture medium on day 12 were measured using ELISA kits (Calbiotech, Spring Valley, CA, USA for E2 and P4, and AnshLabs, Webster, TX, USA for AMH and Androstenedione). The lowest assessment level was 0.1 ng/ml for androstenedione, progesterone, and AMH hormones and 10 ng/ml for 17 β-estradiol. Three separate measurements were taken for each steroid on day 12. The level of hormones was measured in the culture medium obtained from the groups, and a follicle-free culture medium was used as the negative control.

### RNA isolation and real-time PCR

Total RNA of follicles was extracted by TRIzol (TRI reagent, Sigma, Pool, UK) following IVM. A spectrophotometer was used to calculate the optical density (OD) of the samples and the RNA quantity was obtained. Super-script II kit (Fermentase, Sankt leon-rot, Germany) was used to produce the cDNA by Random Hexamer Primers that binds to mRNA as a template, predisposing RNA transcription with dNTPs by a reverse transcriptase enzyme. The real-time PCR process in triplicate using the cDNA from the follicles of different groups was performed using the ABI Prism 7300 Sequence Detector (Applied Biosystems, Foster, USA). The protocol of amplification were 45 cycles with the optimal reaction conditions of activating polymerase enzyme at 95°C for 10 min, denaturation cycle at 95°C for 15 min, and the annealing and elongation at 60°C (based on the primer temperature) for 60 sec (5). Moreover, the β-actin primer was considered as an internal control (Table I). The whole reaction was repeated in 45 cycles and three separate technical replicates were performed for each group.

**Table 1:** Primers used in real-time PCR


**Gene (Mus musculus)**	**Primer (5'-3')**	**Annealing temperature (°C)**	**Product size (bp)**
	Forward: GTCCTACATCTGGCTGAAGTG	61	150
*AMH*	Reverse: GTCCAGGGTATAGCACTAACAG	
		Forward: ATTGCGGAGCTGGAGATG	56	103
*CYP11a1*	Reverse: CTGGAAGTTGAAGAGGATGGG	
		Forward: GAAGGGCAAGGACTTCTCTG	60	153
*CYP17a1*	Reverse: TGAACAGGGCAAAGGTGG	
		Forward: AGCTGAGAAACTGGAAGACTG	59	108
*CYP19a1*	Reverse: GAAGTACAGAGTGACCGACATG	
		Forward: GGGAAAGAGAAACACTGAACAC	60	169
*GNRH*	Reverse: TCTGCCATTTGATCCACCTC	
		Forward: GATTACTGCTCTGGCTCCTAG	61	151
*ACTB*	Reverse: GACTCATCGTACTCCTGCTTG	
	AMH: Anti-Müllerian hormone, CYP11a1: Cytochrome P450 Family 11 Subfamily A Member 1, CYP17a1; Cytochrome P450 Family 17 Subfamily A Member 1, CYP19a1; Cytochrome P450 Family 19 Subfamily A Member 1, GNRH: Gonadotropin-releasing hormone			

### Ethical consideration

All investigations conformed to the ethical and humane principles of research and were approved by the Kermanshah University of Medical Sciences Committee on the Use and Care of Animals, (KUMS.RES.1395.774).

### Statistical analysis

Data were analyzed using the SPSS software (version 22). One-way analysis of variance (ANOVA) test was applied to measure the changes in follicle diameter, degeneration level, antrum formation, hormonal levels, and expression level of the genes obtained among the groups. Data are shown as a mean ± standard deviation with p < 0.0 as a significant level.

## 3. Results 

### Follicle morphology

**Figure 2:**
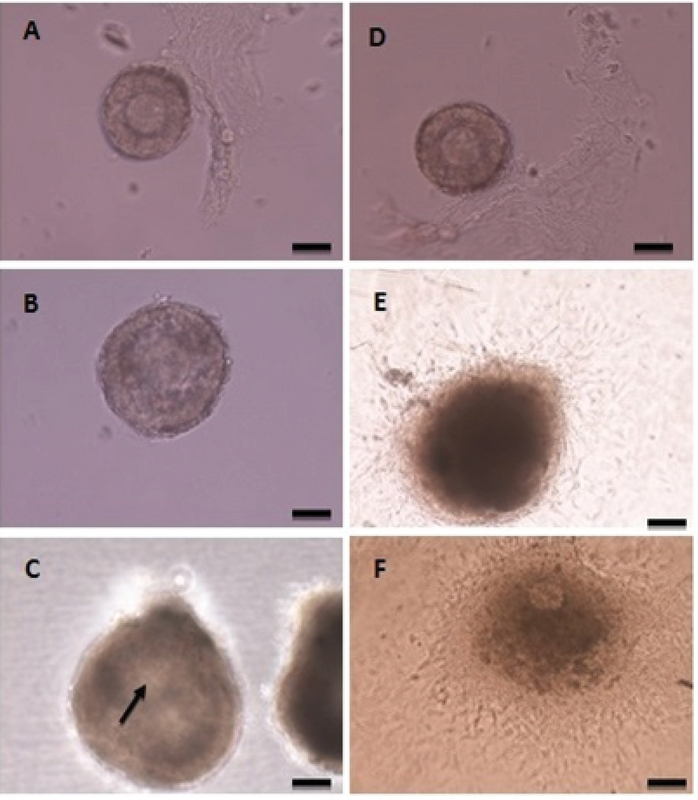
Follicles cultured in 3D medium: (A) day 1, (B) day 6, (C) day 12; Follicles cultured in 2D medium: (D) day 1, (E) day 6, (F) day 12. The 2D culture system is unable to conserve the spherical structure of follicles and the follicles adhere to the plate bottom. Granulosa cells break their basement membrane and distance from the oocyte (F), but the follicles encapsulated with sodium alginate can conserve their 3D follicular structure (C). Arrow mark antrum. Bar = 100 μm.

**Figure 3:**
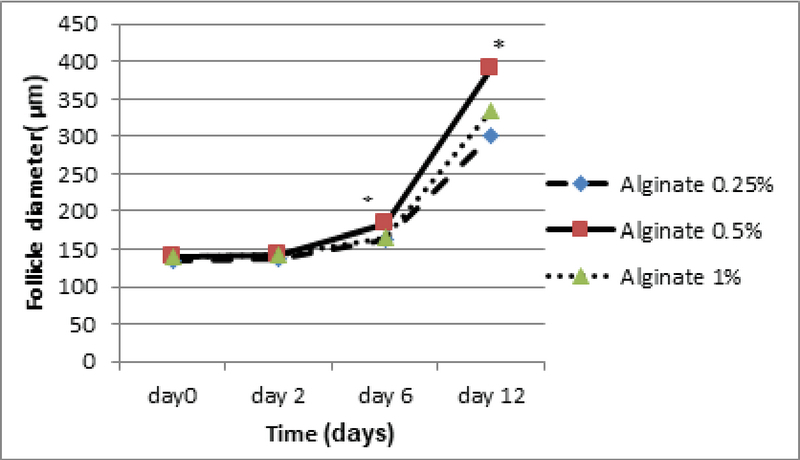
The effect of various concentrations of 3D sodium alginate scaffold (0.25%, 0.5%, and 1%) on follicle diameter on days 2, 6, and 12 of culture. The highest follicle diameter on day 12 is related to the concentration of 0.5%. *: Significant differences with other groups (p = 0.00). Mean ± SD.

**Figure 4:**
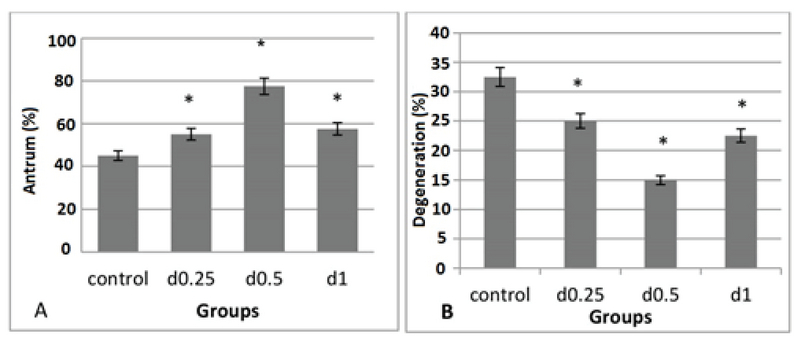
The effect of various concentrations of 3D sodium alginate scaffolds (0.25%, 0.5%, and 1%) and 2D medium (control) on the percentage of antrum formation (A) and the percentage of degenerated follicles (B). The major percentage of antrum formation is related to the concentration of 0.5% and the maximum count of degenerative follicles is related to the 2D medium. *: Significant difference with the control group (p = 0.00).

The follicle diameter was compared at three concentrations of sodium alginate. According to the statistical analysis, no significant differences in follicle diameter among the different concentrations of alginate on day 6 were detected. The largest follicle diameter was seen in the concentration of 0.5% on day 12 (Figure 2), and the follicle diameter in the concentration of 1% was higher than the concentration of 0.25% (p = 0.00; Figure 3). There was a significant increase in the formation of follicular antrum in the concentration of 0.5% compared to the other concentrations and the control group (p = 0.00). However, there was no significant difference between the concentrations of 0.25% and 1% (Figure 4A), as well as the highest and the lowest rates of antral formation were related to the 0.5% concentration (p = 0.00) and control group, respectively. The count of degenerated follicles was significantly decreased at different concentrations of sodium alginate scaffold compared to the control group (p = 0.00). It was significantly different in 0.5% concentration in comparison with the concentrations of 1% and 0.25% (Figure 4B), but no significant alterations were found between the concentrations of 0.25% and 0.5%.

### Hormone levels

**Figure 5:**
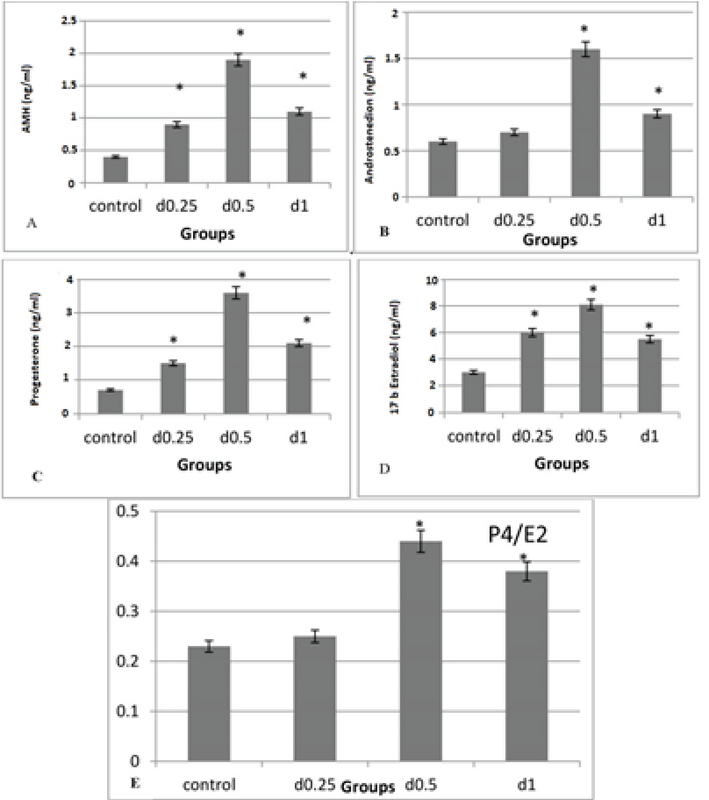
The effect of various concentrations of 3D sodium alginate scaffolds (0.25%, 0.5%, and 1%) and 2D medium (control) on hormonal levels (A), AMH androstenedione (B), progesterone (C), 17β-estradiol (D), and P4/E2 (E). The highest level of these hormones is related to the 0.5% concentration and the lowest level of these hormones in the 2D medium. *: Significant difference with the control group (p = 0.00).

AMH levels in all three concentrations of sodium alginate were significantly higher than the control group (p = 0.00). The highest AMH level was observed at the concentration of 0.5%. Also, there was no significant alteration in hormonal levels between the concentrations of 0.25% and 1% (Figure 5A). The androstenedione level in the 0.5% concentration was higher than other doses and the control group (p = 0.00). There was no significant difference between the concentration of 0.25% and the control group, but the concentration of 1% was significantly higher than the control group (Figure 5B). The progesterone level in all three concentrations of sodium alginate was significantly higher than in the control group (p = 0.00) and the 0.5% concentration was significantly higher than the 0.25% and 1% concentrations (p = 0.00). In addition, the hormone level in the 1% concentration was higher in comparison with the 0.25% concentration (Figure 5C). The 17β-estradiol level was significantly increased at different concentrations of sodium alginate compared to the control group (p = 0.00). The highest level of 17β-estradiol is related to the 0.5% concentration, but there is no significant difference between the concentrations of 1% and 0.25% (Figure 5D). The highest percentage of P4/E2 is significantly related to the 0.5% sodium alginate concentration (p = 0.00; Figure 3 E).

### Gene expression

**Figure 6:**
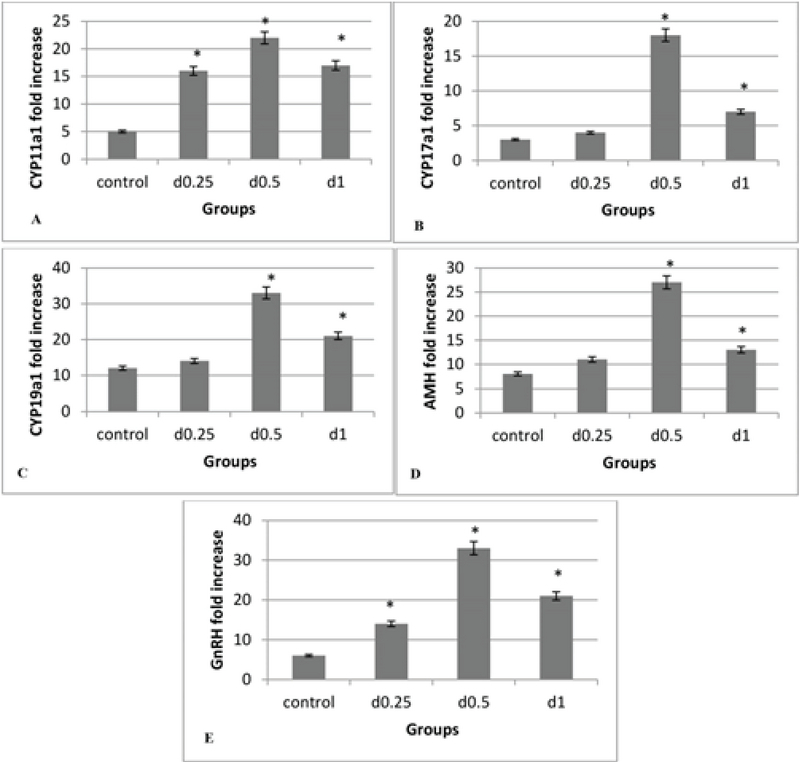
The effect of various concentrations of 3D sodium alginate scaffolds (0.25%, 0.5%, and 1%) and 2D medium (control) on the level of genes expression, (A) *CYP11a1*, (B) *CYP17a1*, (C) *CYP19a1*, (D) *AMH*, and (E) *GnRH*; the highest expression level of these genes is related to the 0.5% concentration and the lowest expression level of these genes in the 2D medium. *: Significant differences with the control group (p = 0.00).

The *CYP11a1* gene expression level in all three concentrations of sodium alginate was greater than in the control group (p = 0.00). Among the different concentrations of sodium alginate, the highest expression level of the gene returned to the 0.5% concentration, but there were no significant differences between the concentrations of 1% and 0.25% (Figure 6A). As shown, the highest expression levels of the *CYP17a1* and *CYP19a1* genes were related to the 0.5% concentration, which is a significant increase compared to the control group (p = 0.00), however, no significant difference between the concentration of 0.25% and the control group (Figures 6B, 6C) were detected. The highest expression levels of *GnRH* and *AMH* genes occurred in the 0.5% concentration (p = 0.00) and the lowest expression levels of these genes were seen in the 2D medium (Figures 6D, 6E).

## 4. Discussion

A number of different cultivation systems with common features and distinct differences have been developed to grow IVG follicles. In order to maintain the cell life and growth in all IVG protocols, it is essential to optimize the supply of nutrients, electrolytes, antioxidants, amino acids, and growth factors to the culture medium (9). The type and concentration of the energy substrate in the base culture medium are important for the culture medium used for IVG and maturation of follicles (10). In this study, the effects of various concentrations of sodium alginate on growth, antrum development, hormonal levels, and expression of steroidogenesis-related genes in the preantral follicles isolated from immature mice ovaries in the culture medium were investigated. In the present study, the follicle diameter on day 6 of the culture in all three groups did not differ significantly. Xu M and colleagues in 2009 investigated the effect of 1% 3D sodium alginate scaffold on bilayered secondary follicles in rats. They reported that the mean diameter of follicles on days 2, 6, and 12 showed no significant differences compared to the control group which is similar to our results on day 6 of the culture (8).

The results on day 12 showed that the follicle diameter in the 0.5% dose was high compared to the other two concentrations and the 3D sodium alginate scaffold showed dose-dependent action. Heise M and colleagues in 2005 conducted a study on the culture of rat preantral follicles in 1% alginate dose. Their results showed that the culture of follicles for 72 hr in the 3D alginate scaffold leads to a significant increase in the diameter of follicles compared to the control group (11), which is consistent with our study. In the present study, the follicle diameter in a dose of 0.25% showed no significant difference in comparison with the control group. Based on our results, the percentage of follicles forming antrum on day 10 at the dose of 0.5% was significantly increased compared to the other two doses and 2D medium. In a study by Ola and colleagues in 2008, the percentage of antrum formation of follicles isolated from rat ovaries in 2D culture was reported to be 38% (12).

West Er and colleagues in 2007 used concentrations of 0.7%, 1.5%, and 3% of alginate hydrogels for follicular culture. They found that the antrum formation in 0.7% concentration was higher than in other concentrations (13). In follicular growth, the follicle diameter increased by the accumulation of water, ions, carbohydrates, and fats. The antrum formation occurs when the follicular diameter reaches from 180 to 250 μm. At this stage, more eggs mature (7). The lowest count of degenerative follicles is seen in the dose of 0.5% and the highest level in the control group because the 2D culture system (non-spherical) is unable to conserve the spherical structure of the follicles and follicles adhere to the bottom of the culture plate and form stack structure. Granulosa cells break their basement membranes and get themselves away from the oocyte, resulting in the loss of cell-cell and cell-matrix interconnections, metabolic impairments, and uncoordinated growth and differentiation of granulosa cells and oocytes (14). The concentration of 0.5% sodium alginate was the optimum for hormone transfer in culture medium for follicle maturation, and 2D medium showed the lowest level of hormone in our study. The sodium alginate is a gelatin material that can be used to encapsulate the follicles providing the surrounding environment of the follicles similar to those in the body and the transfer of hormones and materials needed for follicular maturation occurs easily (15). In our study, the highest level of AMH was seen in the 0.5% concentration, but there was no significant difference between the concentrations of 1% and 0.25%. AMH is the product of granulosa cells in the preantral follicles. The production of AMH causes folliculogenesis, which is regulated by follicle inhibitors to select the dominant follicle, after which the production of AMH is reduced (16).

Xu J and colleagues in 2010 showed that the encapsulation of follicles with alginate in the presence of LH increases the AMH level-up to two weeks, that is, the antrum formation, but has a decreasing trend after the second week (17). In this study, the level of androstenedione hormone was detected more in the concentration of 0.5% compared to the other concentrations and control group. The androstenedione is a weak androgen result of dehydroepiandrosterone (DHEA) and estrogen in the biosynthesis of testosterone. The androstenedione is a precursor for testosterone and other androgens, as well as estrogens like estrogen. The androstenedione is a prehormone with poor androgenic activity. The androgens, as the main androstenedione, are produced by theca cells in response to LH. In the early stages of folliculogenesis, the androgens lead to follicular growth (18). The levels of progesterone and 17β-estradiol in all three concentrations of sodium alginate were increased significantly compared to the control group, and 0.5% concentration was significantly higher than the concentrations of 0.25% and 1%. The progesterone plays a key role in the regulation of granulosa cells and follicular rupture during ovulation. The FSH and LH hormones cause the growth and enlargement of the somatic cells of the follicle. These cells have receptors for these two hormones inducing estrogen production and secretion. The estrogen produced from follicular somatic cells affects themselves and causes further growth of the follicle (19). Xu J and colleagues in 2010 showed that the levels of progesterone, 17β-estradiol, and androstenedione were increased during the 5 wk in alginate scaffolds and in the presence of LH (17).

As seen, the dose of 0.5% sodium alginate had the most favorable concentration. Accordingly, it can be concluded that the 0.25% gel is too thin and the 1% gel is too thick. Thus, the best hormone transfer occurs in 0.5% sodium alginate. Xu M and colleagues in 2009 conducted an in vitro study on the maturation of the monkey follicles encapsulated by two alginate concentrations of 0.25% and 0.5%. Their results showed that the level of androstenedione, 17β-estradiol, and progesterone hormones in the 0.5% concentration was significantly higher than the 0.25% concentration (18). The establishment of in vitro association between the physical properties of the matrix and the features of the tissue is rigorous because the growth of many tissues is impossible due to vascular constraints. Ovarian follicular culture systems provide an ideal condition in which the physical properties of the 3D hydrogel matrix help to develop follicles because the follicles are not vascular and development can be controlled easily and also the follicles mature through the distinct morphological and steroidogenic steps. Sodium alginate hydrogel makes a reticular structure around the follicle allowing the release of essential hormones and proteins for the follicular development. As a result, the hydrogel at low concentrations increases the pore size of the hydrogel that affects the metabolism of the material and the essential macromolecules for the growth and maturation of the follicle and increases the transmission speed of the substances and agents (20). Thus, it can be stated that the concentration of 0.5% sodium alginate induces the most suitable conditions for hormone transfer in the culture medium. The *CYP11a1* gene expression in all three concentrations of sodium alginate is higher than the 2D medium. Among the different concentrations of sodium alginate, the highest gene expression level was related to the 0.5% concentration, but there was no significant difference between the concentrations of 1% and 0.25%. The *CYP11a1* is a mitochondrial enzyme that converts cholesterol into pregnenolone. This is the first reaction of steroidogenesis in all mammals that is responsible for producing various steroid hormones (21). The highest expression level of *CYP17a1* and *CYP19a1* genes were related to the 0.5% concentration. In both genes, there was no significant difference between the concentration of 0.25% and the control group. The *Cyp17a1* is a key enzyme in the pathway of steroidogenesis that produces progesterone, androgens, and estrogens. In particular, the *Cyp17a1* acts on pregnenolone and progesterone (22).

The *CYP19a1* is a key enzyme responsible for estrogen biosynthesis. This gene is a member of the cytochrome P450 family catalyzing many steroidogenesis-related reactions, especially aromatase which is responsible for the aromatization of androgens to estrogen (21). Xu j and colleagues in 2013 identified the *CYP17a1* and *CYP19a1* enzymes in the granulosa and theca area of the follicle using immunohistochemical dye. They showed that the encapsulation of follicles in 3D scaffolds could improve the theca cells and increase the steroid and the steroidogenic functions in the granulosa and theca area of follicles in rats (22). Erin and colleagues in 2009 examined the expression level of *CYP11a1*, *CYP17a1*, and *CYP19a1* genes and finally concluded that the expression level of *CYP11a1* and *CYP17a1* genes at 1.5% sodium alginate concentrations had a significant increase on day 8 of culture compared to the 0.5% concentration, whereas the expression level of the *CYP19a1* gene had a significant increase on day 8 of the culture in the concentration of 0.5% compared to the concentration of 1.5% (21). Among the different concentrations of sodium alginate, the highest and lowest expression levels of *AMH* and *GnRH* genes were 0.5% concentration and 2D medium respectively. The *AMH* gene is a product of granulosa cells, which covers each oocyte and meets its energy. The AMH can also be used as a molecular marker for the relative size of follicles (23).

Silva and colleagues in 2005 investigated the expression of *AMH* gene following 10 days of follicle cultivation and underlined that the expression level of this gene in the in vitro condition was similar to that of in vivo; this gene is expressed in the growing follicle (24). The GnRH stimulates the secretion of FSH and LH, which binds to the receptors on the ovarian follicles, and ultimately induces the secretion of sex hormones, including estrogen and progesterone and a small number of androgens (25). Shahed A and colleagues in 2011 examined the *GnRH* gene in a hamster. Their results showed that the GnRH protein is released during the estrous cycle. This gene is crucial for follicle maturation and steroidogenesis (26). According to the obtained results, it can be concluded that the physical properties of the extracellular matrix, such as mechanical hardness, affect cell behavior in various tissues. Specifically, the physical properties of the hydrogel regulate cellular processes such as cell proliferation, growth factor, and extracellular matrix formation (27).

As it was observed, the concentration of 0.5% sodium alginate was the most favorable concentration. Accordingly, it can be concluded that the 0.25% gel is too thin and the 1% gel is too thick. Thus, the 0.5% sodium alginate gel showed the best concentration for the expression of steroidogenesis-related genes. In the spherical 3D culture system, the spherical structure and physiological relationship within the follicles are well preserved. In addition, the autocrine and paracrine agents secreted from the granulosa cells remain near the oocyte and cause the further effect of these factors on the development of follicles. 3D culture systems are designed to prevent the migration of granulosa cells around the oocyte and adhesion of these cells to the bottom of the culture plate (15). The results of this study showed that different concentrations of alginate hydrogel affect the growth, differentiation, and paracrine signaling among the follicular cell segments. The proper concentration of sodium alginate hydrogel increases follicle growth and causes maturation of follicles, the production of steroid hormones, and the appropriate expression of steroidogenesis-related genes. Our study showed that the concentration of 0.5% sodium alginate can have the greatest effect on the expression of follicle genes.

##  Conflict of Interest

The authors declare that there is no conflict of interest that would prejudice the impartiality of this scientific work.

## References

[1] Lk Książkiewicz

[2] Buratini J., Price C. A. (2011). Follicular somatic cell factors and follicle development. Reproduction, Fertility and Development.

[3] Caldwell A.S.L., Eid S., Kay C.R., Jimenez M., Mcmahon A.C., Desai R., Allan C.M., Smith J.T., Handelsman D.J., Walters Kirsty A. (2015). Haplosufficient Genomic Androgen Receptor Signaling Is Adequate to Protect Female Mice From Induction of Polycystic Ovary Syndrome Features by Prenatal Hyperandrogenization. Endocrinology.

[4] N Wang, Le F, Qt Zhan, L Li, My Dong, Gl Ding

[5] Zhao Shu-Yun, Qiao Jie, Chen Yong-Jian, Liu Ping, Li Jian, Yan Jie (2010). Expression of growth differentiation factor-9 and bone morphogenetic protein-15 in oocytes and cumulus granulosa cells of patients with polycystic ovary syndrome. Fertility and Sterility.

[6] S Abdi, M Salehnia, S Hosseinkhani

[7] Xu Min, West Erin, Shea Lonnie D., Woodruff Teresa K. (2006). Identification of a Stage-Specific Permissive In Vitro Culture Environment for Follicle Growth and Oocyte Development1. Biology of Reproduction.

[8] Xu Min, Banc Anna, Woodruff Teresa K., Shea Lonnie D. (2009). Secondary follicle growth and oocyte maturation by culture in alginate hydrogel following cryopreservation of the ovary or individual follicles. Biotechnology and Bioengineering.

[9] Jin Shi Ying, Lei Lei, Shikanov Ariella, Shea Lonnie D., Woodruff Teresa K. (2010). A novel two-step strategy for in vitro culture of early-stage ovarian follicles in the mouse. Fertility and Sterility.

[10] Pangas Stephanie A., Saudye Hammad, Shea Lonnie D., Woodruff Teresa K. (2003). Novel Approach for the Three-Dimensional Culture of Granulosa Cell–Oocyte Complexes. Tissue Engineering.

[11] M Heise, R Koepsel, Aj Russell, Ea Mcgee

[12] Ola Safiriyu Idowu, Ai Jun-Shu, Liu Jing-He, Wang Qiang, Wang Zhen-Bo, Chen Da-Yuan, Sun Qing-Yuan (2007). Effects of gonadotrophins, growth hormone, and activin A on enzymatically isolated follicle growth, oocyte chromatin organization, and steroid secretion. Molecular Reproduction and Development.

[13] West E, Xu M, Woodruff T, Shea L (2007). Physical properties of alginate hydrogels and their effects on in vitro follicle development. Biomaterials.

[14] Xu J., Lawson M. S., Yeoman R. R., Pau K. Y., Barrett S. L., Zelinski M. B., Stouffer R. L. (2011). Secondary follicle growth and oocyte maturation during encapsulated three-dimensional culture in rhesus monkeys: effects of gonadotrophins, oxygen and fetuin. Human Reproduction.

[15] Kreeger Pamela K., Fernandes Nisha N., Woodruff Teresa K., Shea Lonnie D. (2005). Regulation of Mouse Follicle Development by Follicle-Stimulating Hormone in a Three-Dimensional In Vitro Culture System Is Dependent on Follicle Stage and Dose1. Biology of Reproduction.

[16] Kollmann Zahraa, Bersinger Nick A, Mckinnon Brett D, Schneider Sophie, Mueller Michael D, Von Wolff Michael (2015). Anti-Müllerian hormone and progesterone levels produced by granulosa cells are higher when derived from natural cycle IVF than from conventional gonadotropin-stimulated IVF. Reproductive Biology and Endocrinology.

[17] Xu Jing, Bernuci Marcelo P, Lawson Maralee S, Yeoman Richard R, Fisher Thomas E, Zelinski Mary B, Stouffer Richard L (2010). Survival, growth, and maturation of secondary follicles from prepubertal, young, and older adult rhesus monkeys during encapsulated three-dimensional culture: effects of gonadotropins and insulin. REPRODUCTION.

[18] Xu Min, West-Farrell Erin R., Stouffer Richard L., Shea Lonnie D., Woodruff Teresa K., Zelinski Mary B. (2009). Encapsulated Three-Dimensional Culture Supports Development of Nonhuman Primate Secondary Follicles1. Biology of Reproduction.

[19] Ferreira E.M., Vireque A.A., Adona P.R., Meirelles F.V., Ferriani R.A., Navarro P.A.A.S. (2009). Cytoplasmic maturation of bovine oocytes: Structural and biochemical modifications and acquisition of developmental competence. Theriogenology.

[20] Shikanov Ariella, Xu Min, Woodruff Teresa K., Shea Lonnie D. (2009). Interpenetrating fibrin–alginate matrices for in vitro ovarian follicle development. Biomaterials.

[21] West-Farrell Erin R., Xu Min, Gomberg Monica A., Chow Yee Hoong, Woodruff Teresa K., Shea Lonnie D. (2009). The Mouse Follicle Microenvironment Regulates Antrum Formation and Steroid Production: Alterations in Gene Expression Profiles1. Biology of Reproduction.

[22] Xu J., Lawson M. S., Yeoman R. R., Molskness T. A., Ting A. Y., Stouffer R. L., Zelinski M. B. (2013). Fibrin promotes development and function of macaque primary follicles during encapsulated three-dimensional culture. Human Reproduction.

[23] Rzeszowska Marzena, Leszcz Agnieszka, Putowski Lechosław, Hałabiś Magdalena, Tkaczuk-Włach Joanna, Kotarski Jan, Polak Grzegorz (2016). Anti-Müllerian hormone: structure, properties and appliance. Ginekologia Polska.

[24] Silva J.R.V., Van Den Hurk R., Van Tol H.T.A., Roelen B.A.J., Figueiredo J.R. (2004). Expression of growth differentiation factor 9 (GDF9), bone morphogenetic protein 15 (BMP15), and BMP receptors in the ovaries of goats. Molecular Reproduction and Development.

[25] Choi Jung-Hye, Gilks C. Blake, Auersperg Nelly, Leung Peter C. K. (2006). Immunolocalization of Gonadotropin-Releasing Hormone (GnRH)-I, GnRH-II, and Type I GnRH Receptor during Follicular Development in the Human Ovary. The Journal of Clinical Endocrinology & Metabolism.

[26] Shahed Asha, Young Kelly A. (2011). Intraovarian expression of GnRH-1 and gonadotropin mRNA and protein levels in Siberian hamsters during the estrus cycle and photoperiod induced regression/recrudescence. General and Comparative Endocrinology.

[27] Amorim C. A., Van Langendonckt A., David A., Dolmans M.-M., Donnez J. (2008). Survival of human pre-antral follicles after cryopreservation of ovarian tissue, follicular isolation and in vitro culture in a calcium alginate matrix. Human Reproduction.

